# Application of image processing to evidence for the persistence of the Ivory-billed Woodpecker (*Campephilus principalis*)

**DOI:** 10.1038/s41598-020-71677-5

**Published:** 2020-09-03

**Authors:** Michael D. Collins

**Affiliations:** Naval Research Laboratory, Washington, D.C., 20375 USA

**Keywords:** Scientific data, Statistics, Conservation biology

## Abstract

Image processing is used to enhance apparent field marks in video footage that was obtained during three encounters with birds that were identified in the field as Ivory-billed Woodpeckers (*Campephilus principalis*). Previous analysis of the videos was based on characteristics that are resolved in the raw footage, such as flight path, wing motion, flap rate, behaviors, field marks, and body proportions. Adjusting parameters such as brightness, contrast, and color reveals features consistent with the left dorsal stripe, black leading edges on the dorsal surfaces of the wings, and a red crest that would be consistent with a male of the species. It may be possible to extract additional features from other parts of the videos by applying more advanced processing that allows greater control and analysis of the parameters.

## Introduction

**Figure 1:**
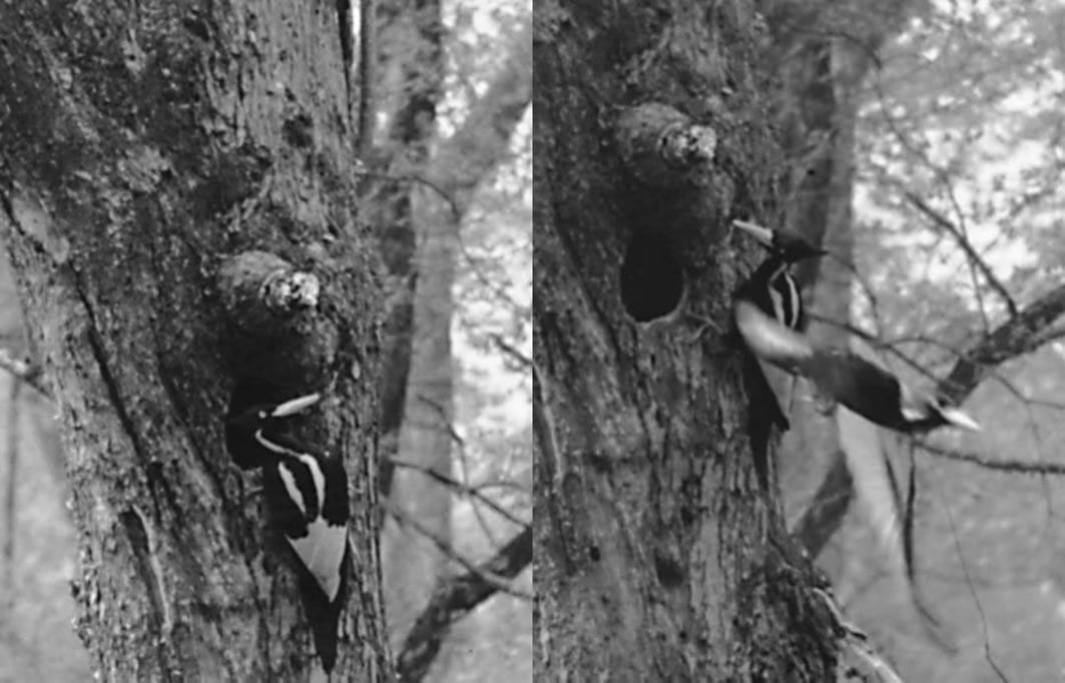
Photos of Ivory-billed Woodpeckers that were obtained by James Tanner at one of the last known nest cavities in 1935. The field marks include two white stripes on the back, a massive white bill, and a white triangular patch that is formed by the white trailing edges on the dorsal surfaces of the wings (which are folded closed on the back). The head of the female is all black. The male has a bright red crest. These photos are in the possession of the U.S. Fish and Wildlife Service and are in the public domain (https://www.fws.gov/ivorybill/photoalbum/).

Elusiveness is often essential to survival for individual animals, but this quality has actually impeded the survival of the Ivory-billed Woodpecker (*Campephilus principalis*)^1–4^, which is so difficult to find and document that it has been presumed extinct only to be rediscovered several times during the past hundred years. In the 1950s, a preserve was temporarily established at a site in Florida following a series of sightings^5^, but there has never existed a sustained conservation program throughout the range of this species. Such programs were established decades ago for the California Condor (*Gymnogyps californianus*), Whooping Crane (*Grus americana*), and Kirtland’s Warbler (*Setophaga kirtlandii*), which might otherwise be extinct by now. High-quality photos, such as those appearing in Fig. 1, and a film were obtained during the only study of the Ivory-billed Woodpecker, which took place in the 1930s near the last known nest sites^1,2^. Numerous sightings were reported during the next several decades, but there were no published reports by ornithologists until 2005^6^. Despite a report the following year by another group of ornithologists^7^, the persistence of the Ivory-billed Woodpecker became controversial when nobody managed to obtain the clear photo that is regarded as the standard form of evidence for documenting birds. More than a decade has passed since those reports came out, but there still does not exist a sustained conservation program for the Ivory-billed Woodpecker, which is perhaps the most imperiled bird in North America. The requirement of ideal evidence as a prerequisite for establishing such a program could have the effect of closing the door to an opportunity to save this iconic species from extinction.

According to an analysis in terms of behavior and habitat, the expected waiting time for obtaining a clear photo of an Ivory-billed Woodpecker is several orders of magnitude greater than it would be for a more typical species of comparable rarity^8^. This result is consistent with the accounts of numerous searchers during the past several decades^3–11^, which suggest that (1) several months of fieldwork per sighting are typically required for this ultra-elusive species and (2) it is nearly impossible to obtain a clear photo without knowing the location of an active nest or roost (nobody has ever managed to do so). It is often possible to establish facts with evidence that is less than ideal. The question of whether the Ivory-billed Woodpecker persists can be resolved with video footage that shows characteristics that are (1) consistent with the Ivory-billed Woodpecker but no other species and (2) sufficient in number to rule out the plausibility of any alternative explanation. Video footage that seems to meet these criteria was obtained during three encounters with birds that were identified in the field as Ivory-billed Woodpeckers^8,12–14^. No flights appear in a film of Ivory-billed Woodpeckers that was obtained in 1935, but historical accounts, such as Audubon’s account of a flight that is “graceful in the extreme”^15^, are suggestive of flight characteristics that might be useful for identification purposes. The videos show several types of flight, all of which seem to be consistent with historical accounts of the Ivory-billed Woodpecker but do not seem to be consistent with the Pileated Woodpecker (*Dryocopus pileatus*), which is the only other large woodpecker that occurs north of the Rio Grande in North America.

Previous analysis of the videos is based on characteristics (such as field marks, flight path, flight speed, wing motion, flap rate, behaviors, and body proportions) that are resolved in the raw footage. The possibility of extracting additional information through image processing is explored here. The improper manipulation of an image may produce misleading results, but the approach used here is to adjust parameters such as color, contrast, and brightness uniformly over every pixel of every frame of each video clip that is considered. This is an objective approach for inspecting video footage for evidence of patterns and colors that are consistent with field marks. The analysis of video footage is a multi-disciplinary task that may benefit from the contributions of experts in disparate fields. Previous analysis of the videos is based on the contributions of an expert on woodpecker flight mechanics, an avian artist who has studied the Ivory-billed Woodpecker, and an expert on applications of statistics and probability. One of the objectives here is to encourage contributions from experts in ornithology and image processing, who may download the videos in raw digital form^16^.

## Methods and materials

The videos were imported from digital videotapes using iMovie 4 and iMovie HD 6.0.3. They were deinterlaced using JES Deinterlacer 3.8.4. Images are processed here using QuickTime Player 7.3.3, GraphicConverter 8.8.3, and GIMP 2.10. Within these applications, it is possible to interpolate and adjust brightness, contrast, color, and other parameters. The simple processing applied here is effective for some cases. With advanced processing techniques that involve greater control and analysis of parameters, experts in image processing might be able to extract additional information.

### The 2006 video

**Figure 2:**
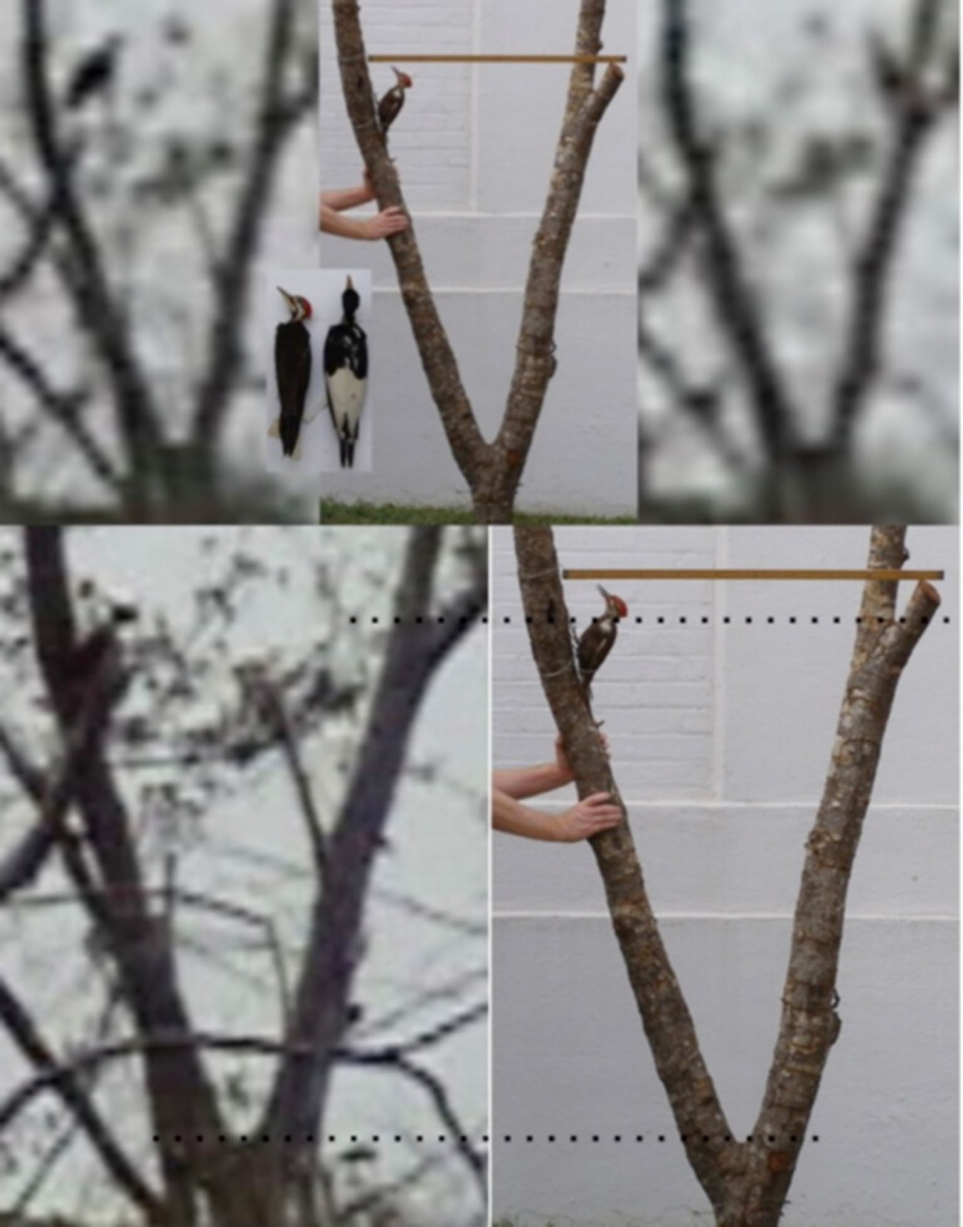
A pileated Woodpecker specimen is mounted on part of the perch tree. Frames from the 2006 video were scaled using forks in the tree (dashed lines). A meter stick is placed at the point where the flight between limbs occurred. The inset shows Pileated Woodpecker and Ivory-billed Woodpecker specimens that were photographed side by side at the National Museum of Natural History. The bird in the video is partially hidden by vegetation in the image on the lower left, but it is fully in view in the images at the top when it took the flight between limbs.

The first video was obtained from a kayak with a Sony DCR-HC36 standard video camera (which captures interlaced video at 720 × 480 pixels) in the Pearl River swamp in Louisiana on February 20, 2006, in an area along English Bayou where there were five sightings that week; the ‘kent’ calls of the Ivory-billed Woodpecker were heard twice during the same period, once coming simultaneously from different directions. The 2006 video shows a large woodpecker perched on a tree, climbing upward, taking a short flight between limbs, and then taking off into a longer flight. Part of the perch tree, which includes two forks that facilitated scaling, was used in the size comparison in Fig. 2; the bird in the video appears to be larger than a Pileated Woodpecker specimen^8^. According to Julie Zickefoose, whose paintings of the Ivory-billed Woodpecker have appeared on the covers of the January 2006 issue of the *Auk* and both editions of Ref.^3^, the “long but fluffy and squared-off crest,” “extremely long, erect head and neck,” “large, long bill,” “bill to head proportions,” “rared-back pose,” “long and thin” wings, “flapping leap” between limbs, and “ponderous and heavy” flight are suggestive of the Ivory-billed Woodpecker but not the Pileated Woodpecker^13^.

### The 2008 video

**Figure 3:**
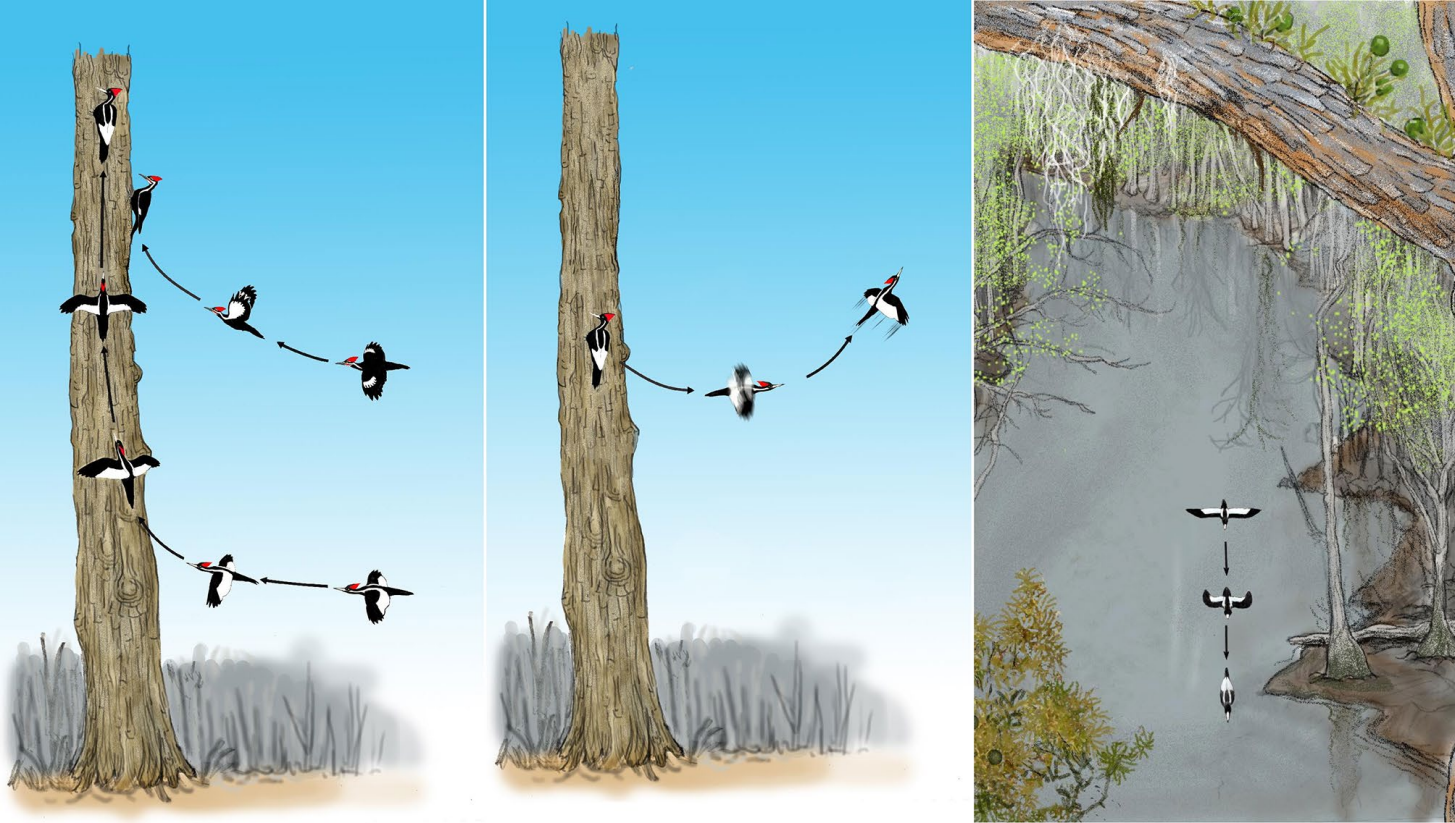
Illustrations of large woodpeckers in flight. Left: The Pileated Woodpecker typically swoops upward a short distance before landing on a surface that faces the direction of approach; the Ivory-billed Woodpecker has long vertical ascents that allow time for maneuvering and landing on surfaces that do not face the direction of approach. Center: An Ivory-billed Woodpecker takes off with rapid wingbeats into a horizontal flight that quickly transitions into an upward swooping flight. Right: Illustration of a flight in the Pearl River swamp on March 29, 2008, that was viewed from 23 m up in a cypress tree. When the wings are folded closed in flight, the dorsal stripes and the white triangular patch have the same appearance as they do for the perched birds in Fig. 1. As discussed in Movie S6 of Ref.^8^, the wings of an Ivory-billed Woodpecker in a historical photo and of the bird in the 2008 video have the swept-back appearance of the wings in the middle image.

A short distance up the same bayou, another video was obtained with the same camera on March 29, 2008, from 23 m up a tree that was used as an observation platform for keeping watch for Ivory-billed Woodpeckers flying over the treetops in the distance. A large bird that flew along the bayou and passed below was identified as an Ivory-billed Woodpecker on the basis of two white stripes on the back and black leading edges and white trailing edges on the dorsal surfaces of the wings (those definitive field marks were observed from an ideal vantage point at close range and nearly directly above). The appearance in the video of the bird, its reflection from the still surface of the bayou, and reference objects made it possible to determine positions along the flight path and obtain estimates of the flight speed and wingspan. The bird in the 2008 video folded its wings closed during the middle of each upstroke as illustrated in Fig. 3. The two large woodpeckers are the only large birds north of the Rio Grande that have this distinctive wing motion, which is clearly resolved in the video. Using an approach that he had previously developed and applied to other woodpeckers^17^, Bret Tobalske, an expert on woodpecker flight mechanics, digitized the horizontal and vertical motions of the wingtips and concluded that the bird in the video is a large woodpecker^13^. The flap rate of the bird in the video is about ten standard deviations greater than the mean flap rate of the Pileated Woodpecker^13^.

Additional characteristics of the bird in the video that are consistent with the Ivory-billed Woodpecker but not the Pileated Woodpecker are the high flight speed, narrow wings, swept back wings, and prominent white patches on the dorsal surfaces of the wings^8,13^. There is one characteristic of the bird in the video that was initially thought to be inconsistent with the Ivory-billed Woodpecker. On the basis of historical accounts of a ‘duck-like’ flight, the Ivory-billed Woodpecker was thought to have a duck-like wing motion in which the wings remain extended throughout the flap cycle. In a series of paintings of the large woodpeckers in flight by Zickefoose^18^, the wings of the Pileated Woodpecker are correctly shown folding closed during the middle of the upstroke; in a proper representation of conventional wisdom at the time, the wings of the Ivory-billed Woodpecker are shown remaining extended throughout the flap cycle (duck-like flaps). An apparent paradox arose during the initial inspection of the video, which revealed an unexpected wing motion. The paradox was resolved after the discovery that a photo from 1939 shows an Ivory-billed Woodpecker in flight at an instant when the wings are nearly folded closed^13^.

### The 2007 video

The other video was obtained with a Sony HDR-HC3 high-definition video camera (which captures interlaced video at 1,440 × 1,080 pixels) that was mounted on kayak paddles^8^ in the Choctawhatchee River swamp in Florida on January 19, 2007, in an area where an ornithologist and his colleagues had recently reported a series of sightings^7^. During an encounter with a pair of birds that were identified as Ivory-billed Woodpeckers on the basis of field marks and remarkable swooping flights, the camera captured a series of events that involve flights, field marks, and other behaviors and characteristics that are consistent with the Ivory-billed Woodpecker but no other species of the region. The analysis of the 2007 video is based in part on the fact that the probability of a series of unlikely events becomes extremely small as the number of events increases^12^. There is a downward swooping takeoff with a long horizontal glide that is consistent with the following account by Audubon^15^: “The transit from one tree to another, even should the distance be as much as a hundred yards, is performed by a single sweep, and the bird appears as if merely swinging from the top of the one tree to that of the other, forming an elegantly curved line.” There are upward swooping landings with long vertical ascents that are not consistent with the Pileated Woodpecker but are consistent with an account by Eckleberry of an Ivory-billed Woodpecker that “alighted with one magnificent upward swoop”^19^.

A long vertical ascent allows time for maneuvering, and the bird appears to rotate about its axis during two of the ascents as illustrated in Fig. 3. In a film of the closely related Magellanic Woodpecker (*Campephilus magellanicus*)^20^, there is maneuvering during a landing with a long vertical ascent. During and after one of the ascents, a woodpecker in the 2007 video shows field marks and body proportions that are consistent with the Ivory-billed Woodpecker but no other species of the region. There is a takeoff into horizontal flight with deep and rapid flaps that are not consistent with the Pileated Woodpecker but are similar to the deep and rapid flaps during a takeoff of the closely related Imperial Woodpecker (*Campephilus imperialis*)^21^. In another event, a woodpecker climbs upward and engages in a series of behaviors that are consistent with the Ivory-billed Woodpecker but no other species of the region, including delivering a blow that produces an audible double knock and taking off with rapid wingbeats into a flight that immediately transitions into an upward swooping flight that is illustrated in Fig. 3.

## Results

### Applications of simple processing

**Figure 4:**
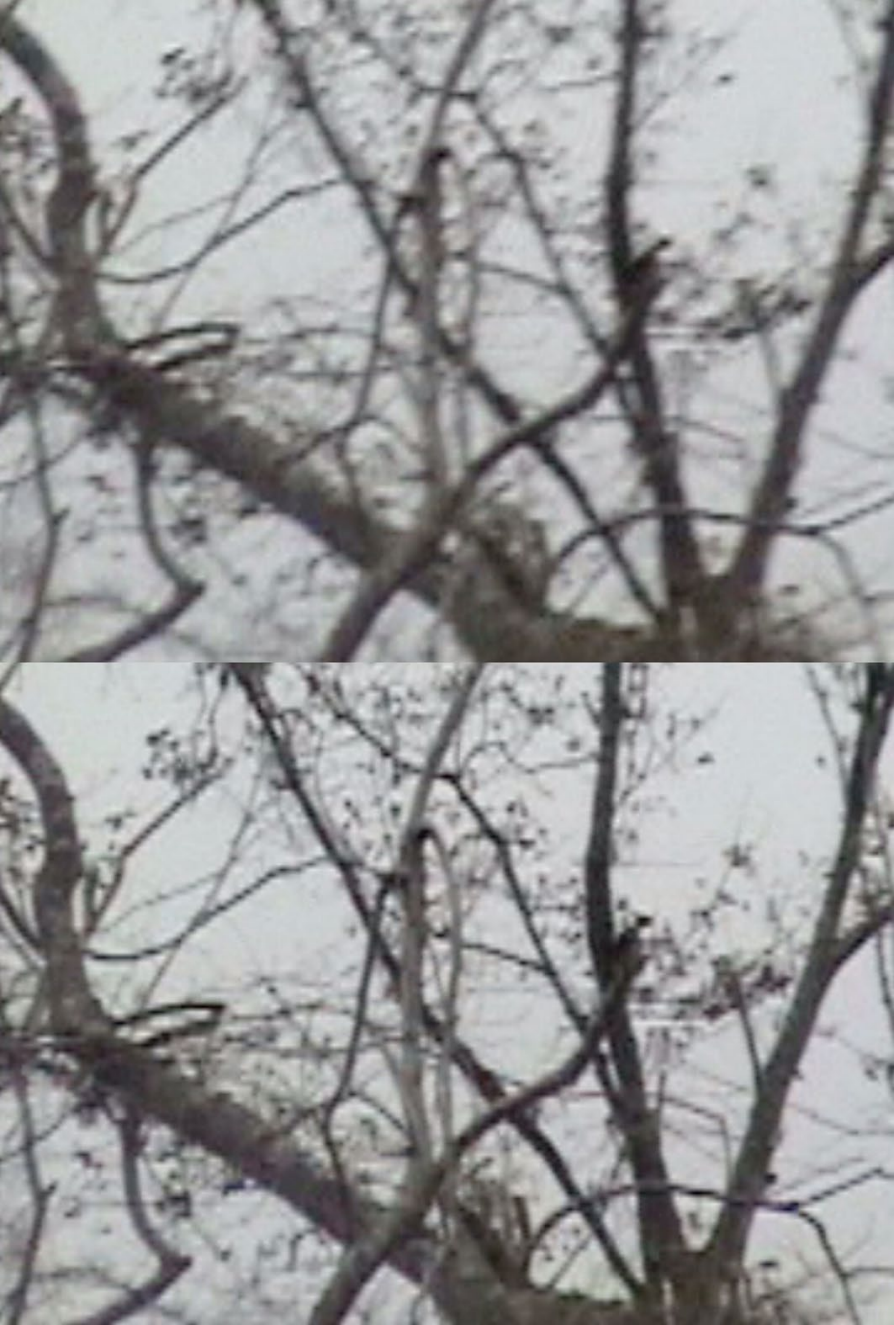
Blurry (above) and sharp (below) frames from the 2006 video.

**Figure 5:**
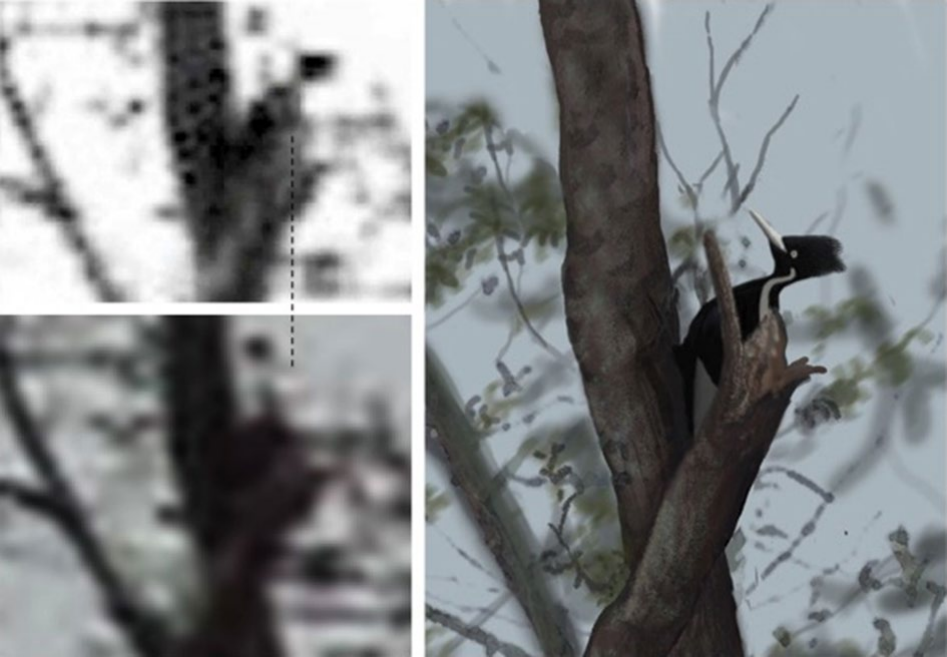
The brightness of an image from the video (upper left) has been adjusted to reveal a feature that is consistent with the left dorsal stripe, which is lined up with the dashed line. Based on an image from the video when the bird was out of view (lower left), it is clear that there is no vegetation at the location of the dorsal stripe feature. An artistic impression appears on the right.

Parts of the 2006 video are blurred due to camera motion, but the bird and surrounding vegetation occasionally come into focus. The images in Fig. 4 were cropped from sharp and blurry frames of the deinterlaced video file 2006p.mov^16^. As shown in Fig. 5, a feature consistent with the left dorsal stripe of an Ivory-billed Woodpecker is revealed when the sharp frame in Fig. 4 is brightened and scaled using cubic interpolation. It was confirmed from other parts of the video that (1) there is no vegetation at the location of the dorsal stripe feature and (2) the faint horizontal white feature across the head corresponds to a thin piece of vegetation. Applying the same approach to other frames of comparable quality did not reveal any sign of a dorsal stripe. The male Ivory-billed Woodpecker and both sexes of the Pileated Woodpecker have red crests. The female Ivory-billed Woodpecker is the only large woodpecker of the region with an all-black crest. As shown in Movies S1 and S2, adjusting the color and contrast revealed a red crest in the woodpecker that gives a double knock in the 2007 video. This is the only event in the videos that is known to show evidence of gender. Although the 2006 video was obtained on an overcast morning, the appearance of green in the vegetation suggests that it may be possible to determine whether or not the crest is red; simple processing with the applications did not reveal any sign of a red crest.

The bird in the 2008 video has white patches on the trailing edges on the dorsal surfaces of the wings that are prominent in some frames. As illustrated in Fig. 3, this field mark is consistent with the Ivory-billed Woodpecker, which also has black leading edges on the dorsal surfaces of the wings. When the bird appears nearly directly below in the video, the black leading edges are difficult to distinguish from the dark mud along the bayou that appears in the background. As discussed in Movie S3, the black leading edges become more apparent when the color, contrast, and brightness are adjusted. As illustrated in Fig. 3, the white trailing edges form a white triangular patch when the wings are folded closed. As discussed in Movie S3, a white feature that is consistent with the white triangular patch appears when the wings are folded closed just after the bird passed nearly directly below.

### Candidates for advanced processing

**Figure 6:**
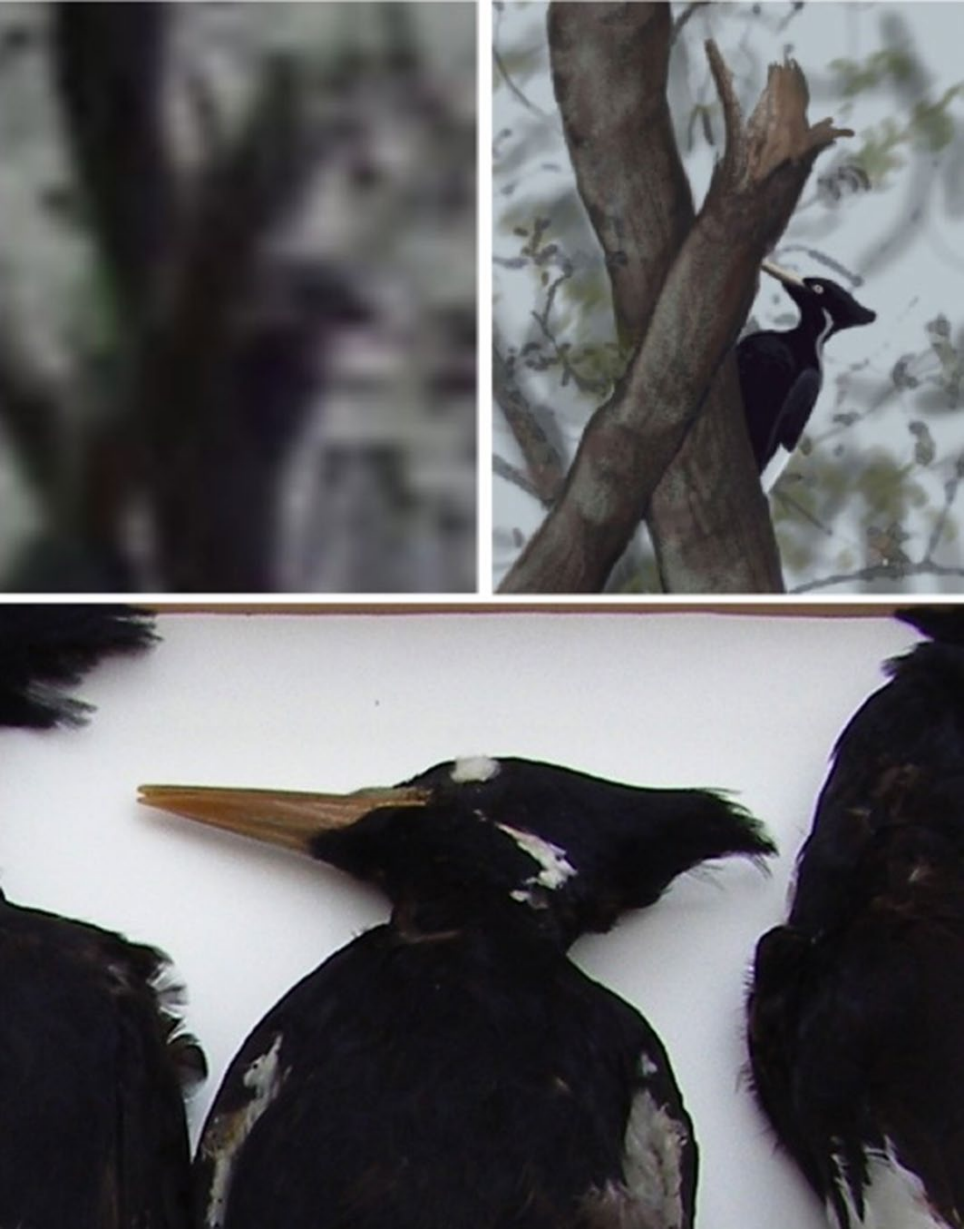
Comparison of a female Ivory-billed Woodpecker specimen (bottom), an image from the video (top left), and an artistic impression (top right). The rounded shape of the top of the head and the shape of the crest of the large woodpecker in the video appear to be consistent with the specimen.

There are several aspects of the 2006 video that might be amenable to advanced processing. It might be possible to determine whether or not the bird in the video has dorsal stripes. Some frames could be analyzed for evidence of field marks on the wings. In the image on the upper right in Fig. 2, there is a trace of a feature during the landing that is suggestive of the white triangular patch that appears on the folded wings of a perched Ivory-billed Woodpecker. In the middle of the flight, there are frames in which the dorsal and ventral surfaces of the right wing are visible. Confirmation that the large woodpecker in the video is larger than a Pileated Woodpecker, as the comparison in Fig. 2 suggests, would be sufficient to confirm the persistence of the Ivory-billed Woodpecker. The crest appears raised in Fig. 2 and relaxed in Fig. 6; the neck, bill, and crest are visible from different angles as the bird climbs and rotates its head and body. Confirmation that the large woodpecker in the video has several features that are consistent with the Ivory-billed Woodpecker but are not consistent with the Pileated Woodpecker, as Zickefoose suggests, would be sufficient to confirm persistence.

**Figure 7:**
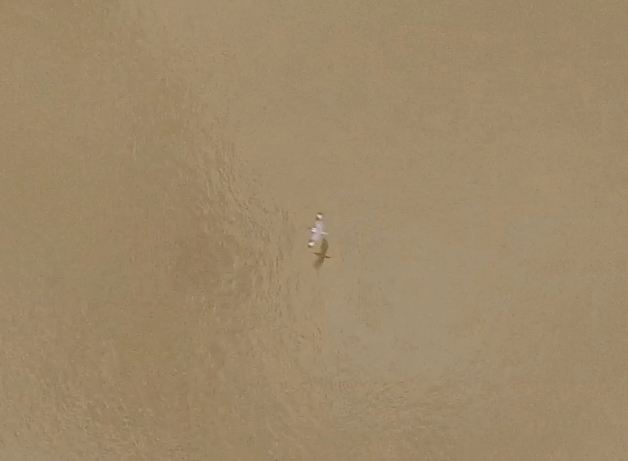
Image of a Belted Kingfisher that was obtained from nearly directly above with a drone.

Parts of the 2008 video might be amenable to advanced processing. There appear to be traces of white patches on the wings while the bird was approaching from down the bayou, but the simple processing used here was not sufficient to confirm them. With advanced processing, it might be possible to obtain better estimates of the aspect ratio of the wings and the wingspan. As discussed in Movie S6 of Ref.^8^, the aspect ratio appears to be consistent with the Ivory-billed Woodpecker but not the Pileated Woodpecker. The wingspan appears to be greater than a 61 cm reference object that appears in a reference photo at the scene^13^. If confirmed, this would rule out the Belted Kingfisher (*Megaceryle alcyon*), which is the third largest species of the region that folds its wings closed (or at least partially closed) during the middle of the upstroke. As shown in Fig. 7, this species has prominent field marks on the dorsal surfaces of the wings, no trace of which is evident after adjusting the color, brightness, and contrast of the 2008 video.

There are several aspects of the 2007 video that might be amenable to advanced processing. During an upward swooping landing that is discussed in Movie S18 of Ref.^8^, the bird appears to have a black belly and mostly white underwings. The two large woodpeckers are the only candidate species with that combination of characteristics; and the long vertical ascent and apparent wing, tail, and body proportions are consistent with the Ivory-billed Woodpecker but not the Pileated Woodpecker. As discussed in Movie S19 of Ref.^8^, there were repeated flashes of white while the bird was climbing after the landing that are consistent with the white triangular patch that appears on the lower back of a perched Ivory-billed Woodpecker. After a landing that follows a long vertical ascent, the bird takes off into a level flight that appears in Movie S4. The body appears to be black, and there is a great deal of white in the wings.

## Discussion

In the raw footage, the large woodpecker in the 2006 video has several characteristics that are consistent with the Ivory-billed Woodpecker but not the Pileated Woodpecker; adjusting the brightness revealed an apparent white stripe on the back, which is consistent with a key field mark of the Ivory-billed Woodpecker; adjusting the color did not reveal any trace of a red crest, the absence of which would be consistent with a female Ivory-billed Woodpecker. In the raw footage, the bird in the 2008 video appears to be a large woodpecker with several characteristics that are consistent with the Ivory-billed Woodpecker but not the Pileated Woodpecker; adjusting the brightness, contrast, and color revealed that the dorsal surfaces of the wings have black leading edges and white trailing edges, which are consistent with the Ivory-billed Woodpecker but not the Pileated Woodpecker; this processing did not reveal any trace of the prominent field marks on the dorsal surfaces of the wings of the Belted Kingfisher. In one of the events in the 2007 video, a woodpecker engages in a series of behaviors that are consistent with the Ivory-billed Woodpecker but not the Pileated Woodpecker; adjusting the color revealed a red crest, which is consistent with a male Ivory-billed Woodpecker. Several candidates for more advanced image processing were identified in the videos. The conservation of the Ivory-billed Woodpecker is a topic of sufficient interest to have been featured on the cover of *Science* (June 3, 2005). This species is worthy of our best and most informed effort to save it from extinction. Video footage that appears to contain conclusive evidence has been archived in raw digital form in order to facilitate independent verification and further analysis.

## Supplementary information

Supplementary Movie S1.

Supplementary Movie S2.

Supplementary Movie S3.

Supplementary Movie S4.
